# A lack of association between BMI and chemoimmunotherapy efficacy in advanced non-small cell lung cancer: Secondary analysis of the IMpower150 and IMpower130 clinical trials

**DOI:** 10.1186/s12885-024-12132-w

**Published:** 2024-03-25

**Authors:** Lee X. Li, Mark A. Socinski, Ganessan Kichenadasse, Christos S. Karapetis, Adel Shahnam, Ross A. McKinnon, Andrew Rowland, Ashley M. Hopkins, Michael J. Sorich

**Affiliations:** 1 College of Medicine and Public Health, Flinders Medical Centre, Flinders University, Bedford Park, Adelaide, SA Australia; 2 AdventHealth Cancer Institute, Orlando, FL USA; 3 Flinders Health and Medical Research Institute, Flinders University, Adelaide, Australia; 4 Flinders Medical Centre, Adelaide, Australia; 5 Department of Medical Oncology, Peter McCallum Cancer Centre, Melbourne, Australia

**Keywords:** Immune checkpoint inhibitor, Chemoimmunotherapy, Body mass index, Non-small-cell lung cancer

## Abstract

**Background:**

Multiple studies have indicated that patients with high body mass index (BMI) may have favourable survival outcomes following treatment with an immune checkpoint inhibitor (ICI). However, this evidence is limited by several factors, notably the minimal evidence from randomised controlled trials (RCTs), the use of categorised BMI with inconsistent cut point definitions, and minimal investigation of contemporary combination ICI therapy. Moreover, whether overweight and obese patients gain a larger benefit from contemporary frontline chemoimmunotherapy in non-small cell lung cancer (NSCLC) is unclear.

**Methods:**

This secondary analysis pooled individual patient data from the intention-to-treat population of the IMpower130 and IMpower150 RCTs comparing chemoimmunotherapy versus chemotherapy. Co-primary outcomes were overall survival (OS) and progression-free survival (PFS). The potentially non-linear relationship between BMI and chemoimmunotherapy treatment effect was evaluated using Multivariable Fractional Polynomial Interaction (MFPI). As a sensitivity analysis, chemoimmunotherapy treatment effect (chemoimmunotherapy versus chemotherapy) on survival was also estimated for each BMI subgroup defined by World Health Organisation classification. Exploratory analyses in the respective chemoimmunotherapy and chemotherapy cohort were undertaken to examine the survival outcomes among BMI subgroups.

**Results:**

A total of 1282 patients were included. From the MFPI analysis, BMI was not significantly associated with chemoimmunotherapy treatment effect with respect to either OS (p = 0.71) or PFS (*p* = 0.35). This was supported by the sensitivity analyses that demonstrated no significant treatment effect improvement in OS/PFS among overweight or obese patients compared to normal weight patients (OS: normal BMI HR = 0.74 95% CI 0.59–0.93, overweight HR = 0.78 95% CI 0.61–1.01, obese HR = 0.84 95% CI 0.59–1.20). Exploratory analyses further highlighted that survival outcomes were not significantly different across BMI subgroups in either the chemoimmunotherapy therapy cohort (Median OS: normal BMI 19.9 months, overweight 17.9 months, and obese 19.5 months, *p* = 0.7) or the chemotherapy cohort (Median OS: normal 14.1 months, overweight 15.9 months, and obese 16.7 months, *p* = 0.7).

**Conclusion:**

There was no association between high BMI (overweight or obese individuals) and enhanced chemoimmunotherapy treatment benefit in front-line treatment of advanced non-squamous NSCLC. This contrasts with previous publications that showed a superior treatment benefit in overweight and obese patients treated with immunotherapy given without chemotherapy.

**Supplementary Information:**

The online version contains supplementary material available at 10.1186/s12885-024-12132-w.

## Background

Over the past decade, immune checkpoint inhibitors (ICIs), especially those targeting programmed cell death 1 (PD-1) or its ligand 1 (PD-L1) such as pembrolizumab, nivolumab, and atezolizumab, have found increasing use either as monotherapy or in combination with chemotherapy (chemoimmunotherapy) for both early and advanced NSCLC [1, 2]. However, while some patients experience positive and durable treatment responses, many do not benefit from ICI treatment [3]. For this reason, much effort has been directed towards identifying predictive markers for ICI efficacy. To this end, various tumour- and host-specific predictors have been investigated [3]. From the latter class, body mass index (BMI), a common and readily available surrogate for obesity status, has been highlighted as a potentially valuable clinical marker for predicting ICI efficacy [4].

Several studies have reported associations between obesity, defined by a high BMI, and favourable survival outcomes in patients with advanced cancer treated with ICIs [5–9]. However, a key limitation of most prior studies is the lack of a control group, which is required to distinguish between the effect of BMI on ICI treatment efficacy and its prognostic impact unrelated to ICI treatment. Notably, of the three prior studies that have included a chemotherapy control group [5–7], only one has based conclusions on randomised controlled trial (RCT) data, which is widely regarded as a key requirement for unbiased evaluation of treatment effects [10, 11]. Of note, this study, which integrated data from two RCTs, suggested that overweight and obese patients with advanced NSCLC may exhibit superior treatment benefits from second- or later-line ICI monotherapy comparative to docetaxel [5]. However, in a recent observational study, BMI was not shown to be a prognostic factor among advanced NSCLC patients treated with first-line chemoimmunotherapy [12]. This highlights a potential deviation from the previously observed association between BMI and ICI treatment efficacy and raises a pivotal question: Does BMI impact the efficacy of ICIs in the context of first-line chemoimmunotherapy – the setting in which ICIs are now often used?

Furthermore, across the literature surrounding ICIs and BMI research, most studies have dichotomised or categorised BMI to define obesity. Such categorisation practices, while perhaps facilitating easier data interpretation, result in a loss of information and undermine statistical power [13]. Additionally, as pointed out by reports of systematic review and meta-analysis [4, 14], the heterogenous results observed in prior studies were partly due to the arbitrarily defined and varied BMI cut points, which have also made comparing and pooling of results across studies difficult, if not impossible.

In response to the highlighted research gaps, this study aims to evaluate the association between BMI, analysed as a continuous measure, and the efficacy of first-line chemoimmunotherapy in patients with advanced NSCLC using individual patient data (IPD) from two RCTs.

## Methods

### Patients

This study was a secondary analysis of anonymised IPD from patients receiving atezolizumab-based chemoimmunotherapy or a control intervention (chemotherapy with or without bevacizumab) as the first-line treatment for advanced non-squamous NSCLC within two open-label phase III RCTs: IMpower130 (NCT02367781, data cut-off 15/3/2018) [15] and IMpower150 (NCT02366143, data cut-off 22/01/2018) [16]. IMpower130 was an RCT of atezolizumab in combination with nab-paclitaxel and carboplatin versus platinum-doublet chemotherapy alone (nab-paclitaxel with carboplatin) for patients with metastatic non-squamous NSCLC [15]. IMpower150 was an RCT comparing atezolizumab plus platinum-doublet chemotherapy (carboplatin with paclitaxel) plus bevacizumab versus platinum-doublet chemotherapy plus bevacizumab for patients with metastatic non-squamous NSCLC [16]. Details of the interventions and procedures, as well as the primary analysis results of the two RCTs were previously published [15, 16]. Data from the Intention-to-treat (ITT) population were pooled.

As with the original analysis of both IMpower130 and IMpower150, the current investigation included the ITT population with wild-type genotype (ITT-WT). Non-WT population with EGFR or ALK genetic alterations were excluded. Further, in line with the primary analysis of IMPOWER150, patients randomised to the atezolizumab plus platinum-doublet chemotherapy without bevacizumab (ACP) arm were excluded from the present investigation.

Secondary analysis of anonymised IPD was deemed as minimal risk research by the Southern Adelaide Local Health Network, Officer for Research and Ethics, and was exempted from review.

### Definitions of variables and outcomes

Baseline BMI at study enrolment was calculated as weight (kg) divided by the square of height (m^2^). Patients with missing height and/or weight information for BMI calculation were excluded from all analyses. The primary analysis utilised BMI as a continuous variable. As a sensitivity analysis, BMI subgroups were identified by the World Health Organisation (WHO) definitions of underweight (< 18.5 kg/m^2^), normal weight (18.5–24.9 kg/m^2^), overweight (25–29.9 kg/m^2^), and obese ($\ge$ 30 kg/m^2^) [17]. As per prior literature investigating the impact of obesity on cancer treatment outcomes and survival [5, 7], the underweight subgroup (4% of total population) was excluded, and the normal BMI was the reference subgroup in exploratory analyses.

The clinical outcomes assessed were overall survival (OS) and progression-free survival (PFS). In both RCTs, PFS was assessed by the investigators according to Response Evaluation Criteria in Solid Tumours (RECIST, version 1.1) [15, 16].

### Statistical analysis

Continuous BMI was modelled in stratified (by RCT) Cox proportional hazards regression by the method of (Multivariable) Fractional Polynomial Interaction (MFPI) [18]. Briefly, MFPI algorithm allows selection of the optimal functional form of BMI to account for any potential non-linear relationships during modelling of BMI-by-treatment interaction. MFPI then performs interaction tests via likelihood ratio (LR) tests on models with and without the chosen interaction term [18]. From the selected interaction model, MFPI estimates the treatment effect (chemoimmunotherapy vs chemotherapy) as hazard ratios (HRs) at each point of BMI within the range of interest (18.5-40kg/m^2^) [18]. These treatment effect estimates are presented as pointwise HRs with the corresponding 95% confidence intervals (CIs), and are plotted against BMI for visualisation of any potential differential treatment effect [18]. This plot serves as an inferential tool complementary to the interaction test described above [18]. A straight line parallel to the x-axis indicates a lack of effect, whereas a non-constant line—increasing, decreasing, or curved—signals potential differential treatment effects [18]. Further technical details of the MFPI analysis are summarised in additional file 1.

As a sensitivity analysis, chemoimmunotherapy treatment effects were estimated for BMI subgroups utilising Cox proportional hazards regression stratified by RCT with a BMI-by-treatment interaction term. The BMI subgroup treatment effects were presented using forest plots. Survival by treatment in respective BMI subgroups was estimated and plotted using the Kaplan Meier product limit method.

Exploratory analyses on differences in survival prognosis between BMI subgroups were undertaken in the chemoimmunotherapy cohort and chemotherapy cohort separately. Using normal weight category as the reference group, hazards ratio (HRs) and the corresponding 95% CIs were estimated in Cox proportional hazards regression models stratified by RCT.

Median follow up was estimated using the reverse Kaplan Meier method. Analyses related to MFPI were performed using Stata (version 17.0, StataCorp). All other analyses were performed using R (V4.2.3, R Foundation for Statistical Computing). All statistical tests were two-sided, and a p-value < 0.05 were considered statistically significant.

## Results

### Patient and cohort characteristics

The pooled ITT populations consisted of 1925 patients, of which 1282 patients met the inclusion criteria. Two-hundred and one (10%) non-WT patients, 47 (2%) patients with missing height and/or weight data, 68 (4%) underweight patients, and 349 (18%) patients randomised to ACP arm in IMpower150 were excluded. Of the included patients, 761 (59%) received atezolizumab-containing chemoimmunotherapy, and 521 (41%) received chemotherapy. With respect to BMI status, 590 (46%) were normal weight, 455 (35%) were overweight, and 237 (19%) were obese at study entry.

The demographic and baseline clinical characteristics of the analysis cohort are summarised by RCT, by intervention, as well as by BMI category in each intervention cohort (Supplementary Table 1- 4, see additional file 2). The median follow-up of all included patients was 19.6 months (interquartile range (IQR): 15.3 – 23.4 months), and the median follow-up in the IMpower130 and IMpower150 cohorts were 18.6 months (IQR: 15.2 – 23.4 months) and 19.8 months (IQR: 15.4 – 23.3 months) respectively.

### BMI and chemoimmunotherapy efficacy

**Fig. 1:**
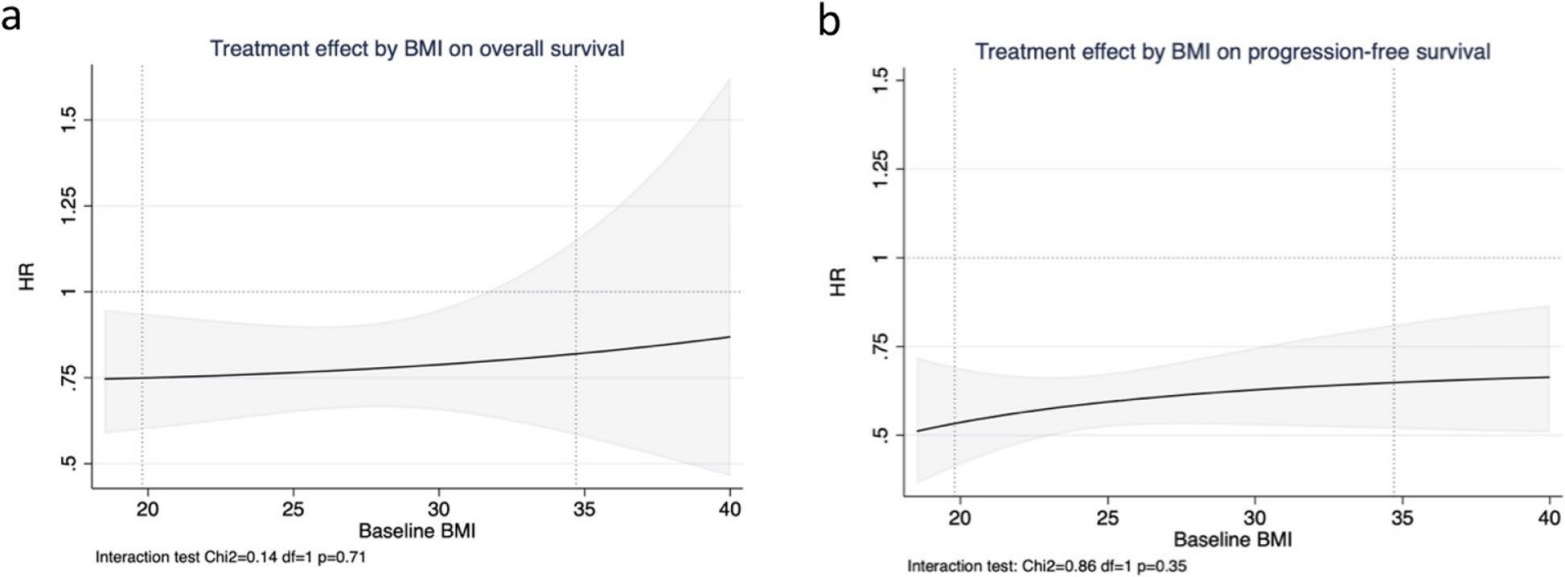
Treatment effect estimates (chemoimmunotherapy versus chemotherapy) with continuous BMI by the method of Multivariate Fractional Polynomial Interaction with Cox proportional-hazard regression models stratified by trial for overall survival (**a**) and progression-free survival (**b**). The shaded area is the 95% CIs of the treatment effect estimated as hazard ratios (HRs) (black solid line). Vertical dotted lines mark the 5^th^ and 95^th^ percentile of BMI distribution of the pooled analysis cohort, at 19.8 kg/m^2^ and 34.7 kg/m^2^ respectively

**Fig. 2:**
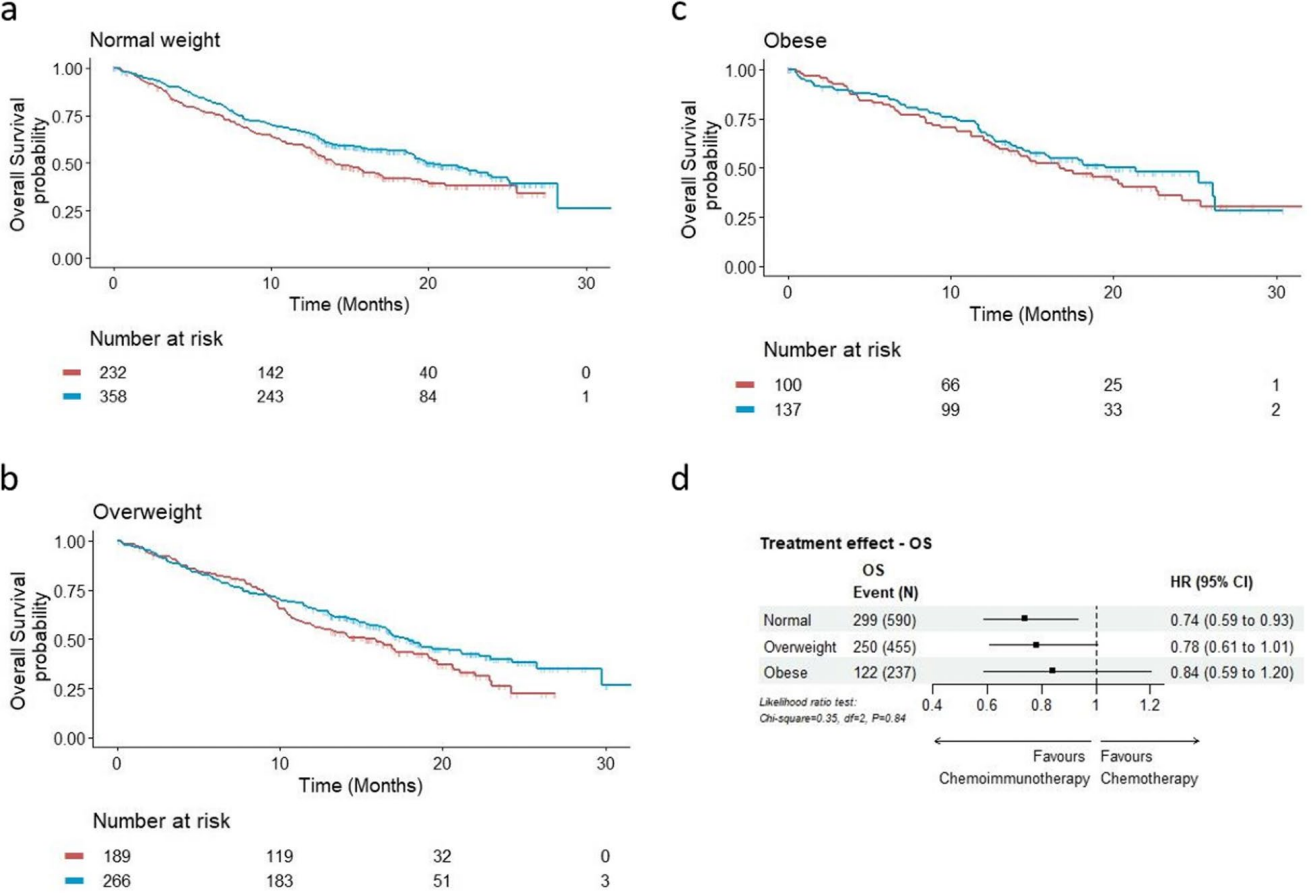
Sensitivity analysis. Overall survival^†^ by treatment in the normal weight (**a**), overweight (**b**), and obese (**c**) cohort, with chemoimmunotherapy treatment effect estimates^††^ by BMI category (**d**) ^†^Kaplan-Meier product limit estimates were derived from the pooled cohort of two RCTs. Blue: chemoimmunotherapy; Red: chemotherapy ^††^Forest plot estimates (chemoimmunotherapy versus chemotherapy) were derived from stratified (by RCT) Cox proportional hazards regression models with a BMI-by-treatment interaction term

**Fig. 3:**
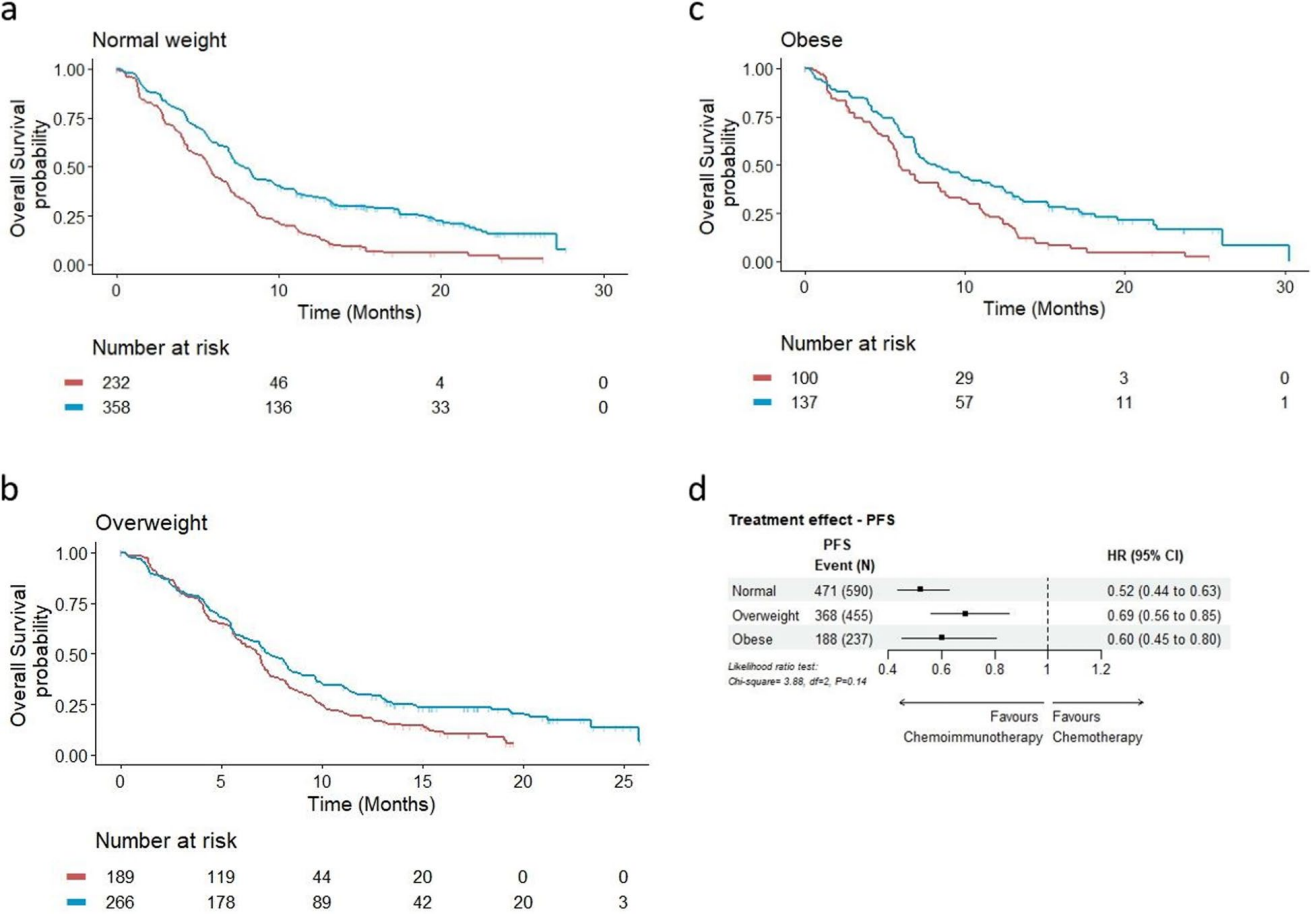
Sensitivity analysis. Progression-free survival^†^ by treatment in the normal weight (**a**), overweight (**b**), and obese (**c**) cohort, with chemoimmunotherapy treatment effect estimates^††^ by BMI category (**d**) ^†^Kaplan-Meier product limit estimates were derived from the pooled cohort of two RCTs. Blue: chemoimmunotherapy; Red: chemotherapy ^††^Forest plot estimates (chemoimmunotherapy versus chemotherapy) were derived from stratified (by RCT) Cox proportional hazards regression models with a BMI-by-treatment interaction term

In the primary analysis with continuous BMI as the predictor variable, no significant changes in treatment effect estimates were observed across the BMI range for either OS (Fig. 1a) or PFS (Fig. 1b), as demonstrated by the constant line parallel to x-axis. Interaction tests (LR test) provided further evidence for such a lack of differential treatment effect (OS: *p* = 0.71; PFS: *p* = 0.35). Figures 2 and 3 show the KM plots and forest plots from the sensitivity analysis, summarising the chemoimmunotherapy treatment effect estimates by BMI categories for OS and PFS outcomes. Like the results observed from the primary analysis, chemoimmunotherapy treatment effect did not significantly differ across BMI subgroups, either for OS (Fig. 2d. Normal: HR = 0.74 95% CI 0.59–0.93; Overweight: HR = 0.78 95% CI 0.61–1.01; Obese: HR = 0.84 95% CI 0.59–1.20) or PFS (Fig. 3d. Normal: HR = 0.52 95% CI 0.44–0.63; Overweight: HR = 0.69 95% CI 0.56–0.85; Obese: HR = 0.60 95% CI 0.45–0.80).

### Exploratory analysis of prognostic associations within specific treatment cohorts

Within the chemoimmunotherapy-treated cohort, no significant prognostic association was observed between baseline BMI subgroups and OS (log-rank test: *p* = 0.7) or PFS (log-rank test: *p* = 0.4) outcomes (Supplementary Fig. 1a and 1b, see additional file 2). The median OS was 19.9 months (95% CI 18.7–24.0) for the normal BMI group, 17.9 months (95% CI 16.3–22.2) for the overweight group, and 19.5 months (95% CI 14.9-n/a) for the obese group. Similarly, in the chemotherapy-treated cohort, no significant prognostic association was observed between baseline BMI subgroups and survival outcomes (log-rank test: OS, *p* = 0.7; PFS, *p* = 0.2; Supplementary Fig. 1c and 1d, see additional file 2). The median OS was 14.1 months (95% CI 12.8–17.1) for the normal BMI group, 15.9 months (95% CI 12.1–19.1) for the overweight group, and 16.7 months (95% CI 13.5–22.8) for the obese group. Median PFS estimates in the chemoimmunotherapy and chemotherapy cohort are reported in supplementary Figure 1 (see additional file 2).

## Discussion

To our knowledge, this investigation is the first to evaluate the association between baseline BMI and chemoimmunotherapy treatment efficacy utilising data from RCTs. Our findings indicate that individuals with a high baseline BMI do not gain any larger treatment benefit from chemoimmunotherapy in the setting of front-line treatment of advanced NSCLC.

Our primary analysis focused on examining BMI as a continuous measure – an unconventional approach in contrast to prior literature. We demonstrated the feasibility of such analyses and presented them as alternative to the traditional subgroup analysis, in the hope of encouraging a wider adaptation of this approach. The perils of dichotomising or categorising a continuous variable during data analysis have been well-described in both reports with robust statistical reasoning [13, 19], and editorial commentaries with lay terms and examples that are accessible to a wider audience [20, 21]. The main concern with dichotomising or categorising a continuous variable is two-fold. Firstly, it wastes data, and thus leads to a loss of statistical power, and, secondly, the cut points are often arbitrary and biologically implausible [13, 20].

The key finding of our study differs from the two prior BMI analyses that evaluated both ICI monotherapy and chemotherapy patient cohorts [5, 6]. Both prior studies were suggestive of a larger treatment benefit (compared to chemotherapy) from first-line or second/later-line ICI monotherapy among patients who had high baseline BMIs. In contrast, the current study evaluated chemoimmunotherapy, and it is possible that the differences may be due to the addition of chemotherapy to ICI, which is known to enhance tumour antigenicity and improve survival outcomes in NSCLC [22]. As noted, Cortellini and colleagues have recently reported that BMI was not associated with prognosis in a cohort of advanced NSCLC patients treated with chemoimmunotherapy [12]. While this study did not include a chemotherapy control group and was therefore unable to estimate chemoimmunotherapy treatment benefit, the results are consistent with our findings from the primary and exploratory analyses. Importantly, countering against the much-reported phenomenon of the “obesity paradox” in cancer epidemiology [23, 24], our findings serve as cautionary evidence against using BMI as a stratification factor in RCTs or as a selection factor for chemoimmunotherapy in clinical practice.

Our study has several strengths, most notable of which is the evaluation of BMI as a continuous variable and thus avoiding potential issues arising from using cut points. Other strengths included randomised treatment allocation that facilitates evaluation of treatment efficacy, high quality data, and a well-defined population. However, the clinical trials cohorts are more highly selected than real world populations and may have some limitations of generalisation. Additionally, this was a post-hoc analysis, and overweight/obesity was defined by BMI, which is an imperfect measure of obesity. Specifically, BMI alone does not provide information on body fat and muscle composition, thus it is not a marker for conditions such as sarcopenic obesity – a body composition type associated with poorer cancer survival outcomes [25]. Moreover, weight loss as part of paraneoplastic syndrome during the period leading up to diagnosis and/or treatment is an established prognostic factor in lung cancers [26, 27], and may be related to immunotherapy efficacy. Clinical trials, however, typically only record a single baseline weight measurement, and do not capture any pre-diagnosis information. It was therefore not possible to evaluate the relationship between pre-diagnosis or pre-treatment weight loss and chemoimmunotherapy treatment efficacy. Future studies would benefit from longitudinal BMI measurements, particularly measurements from the pre-diagnosis and pre-treatment periods, in conjunction with other anthropometric measurements of obesity. Lastly, our study included data from RCTs on atezolizumab-containing chemoimmunotherapy for advanced NSCLC. To further advance our knowledge on BMI’s association with ICI efficacy, a detailed meta-analysis of all major trials on key ICIs in their respective contemporary roles in cancer treatment is warranted. This is, however, a task presently challenged by limitations in clinical trial data sharing [28].

## Conclusions

In conclusion, there was no evidence that individuals with a higher baseline BMI gained any larger treatment benefit from chemoimmunotherapy in front-line treatment of advanced non-squamous NSCLC. This challenges the notion of BMI as an emerging clinical marker for ICI treatment efficacy and invites future research to further our understanding of the complex interplay between obesity and ICI treatment efficacy.

### Supplementary Information

**Additional file 1.** Multivariable fractional polynomial interaction (MFPI) analysis

**Additional file 2.** Supplementary figure; Supplementary table 1–4

## Data Availability

This publication is based on research using data from data contributor, Roche, that has been made available through Vivli, Inc. Vivli has not contributed to or approved, and is not in any way responsible for, the contents of this publication.

## Abbreviations

BMI: Body mass index
CI: Confidence intervals
HR: Hazards ratio
ICI: Immune checkpoint inhibitor
IPD: Individual patient data
IQR: Interquartile range
ITT: Intention-to-treat
LR: Likelihood ratio test
MFPI: Multivariable Fractional Polynomial Interaction
NSLCL: Non-small cell lung cancer
OS: Overall survival
PFS: Progression-free survival
RCT: Randomised controlled trials
WHO: World Health Organisation
WT: Wild-type genotype
