# Supplementary material for: A lack of association between BMI and chemoimmunotherapy efficacy in advanced non-small cell lung cancer: Secondary analysis of the IMpower150 and IMpower130 clinical trials
Source: BMC Cancer. 2024 Mar 25;24:379. doi: 10.1186/s12885-024-12132-w (PMC10964615; doi:10.1186/s12885-024-12132-w)
Supplement: Supplementary file 1 — Additional file 1. Multivariable fractional polynomial interaction (MFPI) analysis [file 12885_2024_12132_MOESM1_ESM.docx]

# Multivariable Fractional Polynomial Interaction (MFPI) analysis

The central element of MFPI is the use of fractional polynomial (FP) functions to capture non-linear relationships. Polynomial-based transformation of a continuous variable, such as by logarithmic or quadratic transformation, is often deployed as the first port-of-call by analysts confronting the issues of covariate non-linearity during regression modelling (Royston & Altman, 1994). However, such polynomial transformations are often applied on an ad-hoc basis, and suffer from the issue of a limited family of shapes particularly in lower order polynomials (Royston & Altman, 1994). To address these limitations, Royston and Altman (1994) proposed fractional polynomials (FP) – a modelling procedure that includes an extended family of curves defined by a pre-specified small set of power terms. FP, therefore, formalises the practice of polynomial transformation in modelling of non-linear continuous covariates, and is applied in MFPI procedures.

### MFPI in primary analysis

In order to detect treatment effect modification by a continuous covariate, MFPI was developed extending the FP methods by inclusion of covariate-by-treatment interaction terms in the MFPI algorithm. Detailed descriptions of the MFPI procedures by the developers are available (Royston & Sauerbrei, 2004, 2009). In the present analysis, we applied the first-degree FP and flex3 (FP1 Flex3) variant of the MFPI algorithm in stratified Cox proportional hazards models. It has been demonstrated in simulation studies that, if no particular functional relationship (i.e non-linear relationship) is expected from prior evidence, such as the case of present investigation of BMI and immunotherapy treatment effect, the FP1 flex3 variant provides a good flexibility and preservation of nominal significance levels (Royston & Sauerbrei, 2014).

FP functions with one power term are known as FP1. The optimal power to describe BMI is chosen from the set S=(−2, −1, −0.5, 0, 0.5, 1, 2, 3) and zero denotes log $Z$. Thus, there are eight candidate FP1 functions to describe BMI, including the linear function (BMI untransformed in its original scale). The optimal FP1 function was chosen with the highest log-likelihood (or lowest deviance) in the stratified (by trial) Cox Proportional-Hazard models (CPH).

From the CPH with the optimal BMI functional form chosen, treatment effect was estimated as HRs by comparing the predicted risk scores from the treatment group against that of the control group at each point of BMI of interest. These pointwise treatment effect HRs were then plotted against BMI for inference. A straight line parallel to the x-axis in these plots indicates a lack of differential treatment effect, whereas a non-constant line, often increasing, decreasing, or curved, signals potential prognostic or predictive effects (Sauerbrei & Royston, 2022). Analogous to categorical BMI analysis, the 95% CI indicates estimation (un)certainty. In addition, values below or above the line of no effect (HR=1) represent beneficial or harmful treatment effect respectively.

Lastly, it should be noted that FPs, and thus MFPI procedures, are parametric methods with functions exhibiting global-influence (Patrick Royston, 2008b). Due to this feature, outlying observations may exert undue influence on the overall model fit (Patrick Royston, 2008b). To minimise this, BMI values above 99% centile were replaced by the value at 99% centile in all MFPI analyses (note that underweight patients were excluded from analysis) (Kasenda et al., 2016; Patrick Royston, 2008a). While this global function feature may be considered a drawback, it allows a greater transferability for future applications compared to other methods such as regression splines, smoothing splines, and kernel-based smoothers (Patrick Royston, 2008b).

### References:

Kasenda, B., Sauerbrei, W., Royston, P., Mercat, A., Slutsky, A. S., Cook, D., Guyatt, G. H., Brochard, L., Richard, J.-C. M., Stewart, T. E., Meade, M., & Briel, M. (2016). Multivariable fractional polynomial interaction to investigate continuous effect modifiers in a meta-analysis on higher versus lower PEEP for patients with ARDS. *BMJ open, 6*(9), e011148-e011148. <https://doi.org/10.1136/bmjopen-2016-011148>

Patrick Royston, W. S. (2008a). Chapter 3. Handling Categorical and Continuous Predictors. Multivariable Model - Building: A Pragmatic Approach to Regression Anaylsis based on Fractional Polynomials for Modelling Continuous Variables. In *Wiley Series in Probability and Statistics*. Wiley.

Patrick Royston, W. S. (2008b). *Multivariable Model - Building: A Pragmatic Approach to Regression Anaylsis based on Fractional Polynomials for Modelling Continuous Variables*. Wiley.

Royston, P., & Altman, D. G. (1994). Regression Using Fractional Polynomials of Continuous Covariates: Parsimonious Parametric Modelling. *Applied Statistics, 43*(3), 429-467. <https://doi.org/10.2307/2986270>

Royston, P., & Sauerbrei, W. (2004). A new approach to modelling interactions between treatment and continuous covariates in clinical trials by using fractional polynomials. *Statistics in medicine, 23*(16), 2509-2525. <https://doi.org/10.1002/sim.1815>

Royston, P., & Sauerbrei, W. (2009). Two Techniques for Investigating Interactions between Treatment and Continuous Covariates in Clinical Trials. *The Stata journal, 9*(2), 230-251. <https://doi.org/10.1177/1536867X0900900204>

Royston, P., & Sauerbrei, W. (2014). Interaction of treatment with a continuous variable: simulation study of power for several methods of analysis. *Statistics in medicine, 33*(27), 4695-4708. <https://doi.org/10.1002/sim.6308>

Sauerbrei, W., & Royston, P. (2022). Investigating treatment-effect modification by a continuous covariate in IPD meta-analysis: an approach using fractional polynomials. *BMC medical research methodology, 22*(1), 98-98. <https://doi.org/10.1186/s12874-022-01516-w>
