# Supplementary material for: A lack of association between BMI and chemoimmunotherapy efficacy in advanced non-small cell lung cancer: Secondary analysis of the IMpower150 and IMpower130 clinical trials
Source: BMC Cancer. 2024 Mar 25;24:379. doi: 10.1186/s12885-024-12132-w (PMC10964615; doi:10.1186/s12885-024-12132-w)
Supplement: Supplementary file 2 — Additional file 2. Supplementary figure; Supplementary table 1–4 [file 12885_2024_12132_MOESM2_ESM.docx]

# Supplementary figure

Supplementary Figure 1: Overall survival^†^ (a and c) and progression-free survival^†§^ (b and d) by BMI category in the chemoimmunotherapy (a and b) and chemotherapy cohort (c and d) with survival outcome comparisons^††^ between BMI categories.

| a  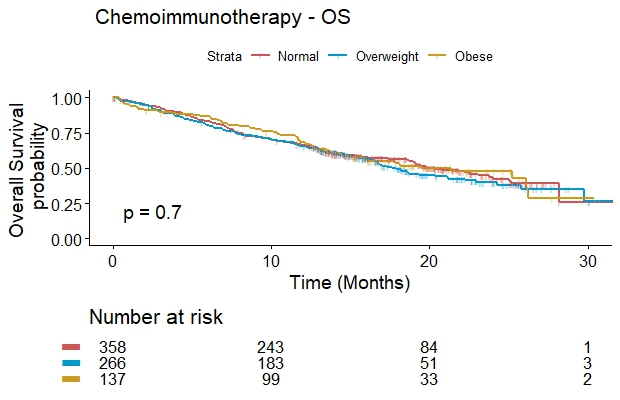  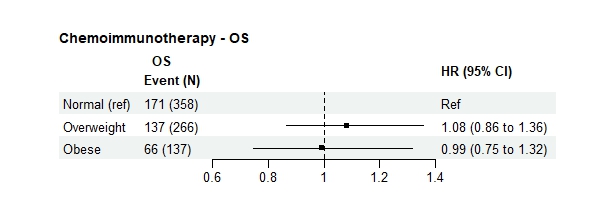 | c  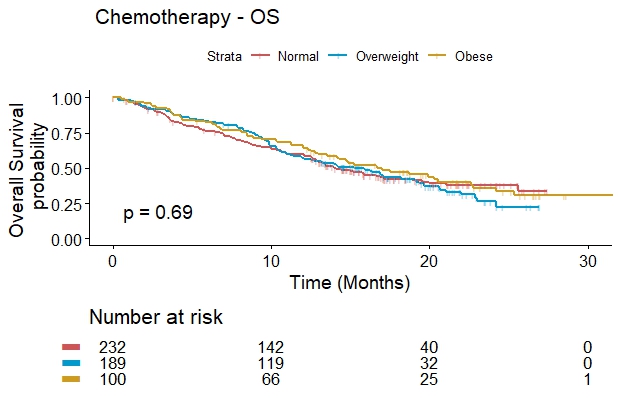  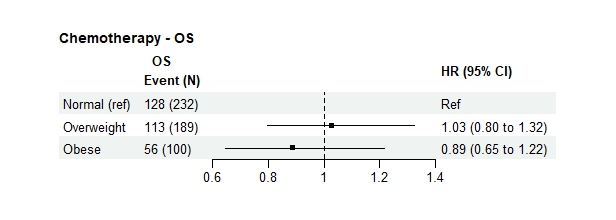 |
| --- | --- |
| b  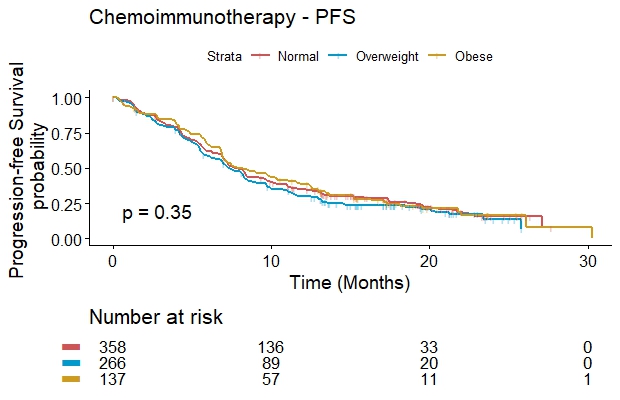  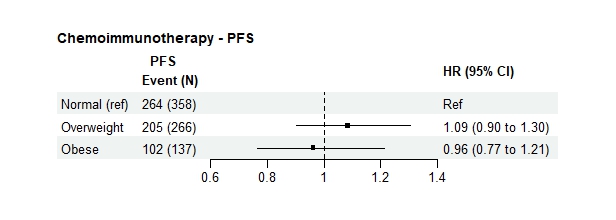 | d  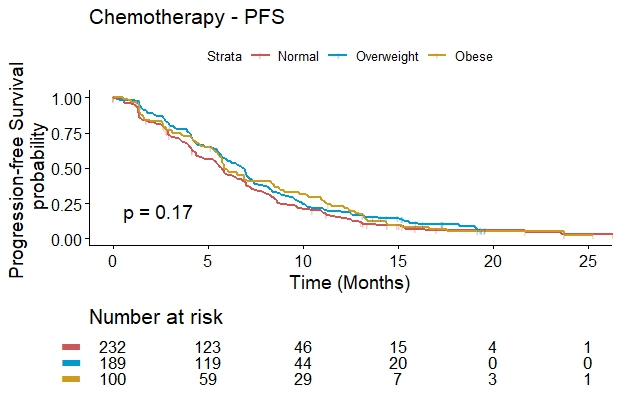  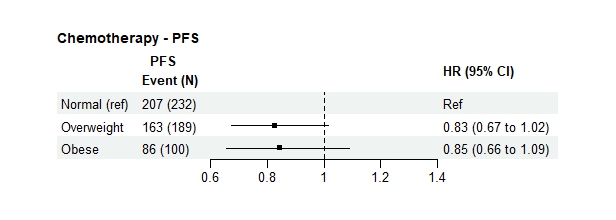 |
| ^†^Kaplan-Meier product limit estimates were derived from the pooled cohort of two RCTs. P-values were derived from log-rank tests.  ^††^Forest plot estimates were derived from Cox proportional hazards regression models stratified by RCT.  ^§^ Median PFS in chemoimmunotherapy cohort: normal weight 7.9 (95% CI 7.1-8.4) months, overweight 7.3 (95% CI 6.9-8.3) months, and obese 8.2 (95% CI 7.0-11.6) months.  ^§^ Median PFS in chemotherapy cohort: normal weight were 5.7 (95% CI 5.0-6.5) months, overweight 6.8 (95% CI 5.9-7.2) months, and obese 5.9 (95% CI 5.6-8.4) months. | |

# Supplementary tables

Supplementary Table 1: Patient characteristic data by clinical trial.

|  | | | |
| --- | --- | --- | --- |
|  | **Total No. 1,282** | **IMPOWER130 No. 648** | **IMPOWER150 No. 634** |
| Randomised Arm | | | |
| Atezolizumab + Bevacizumab + Carboplatin + Paclitaxel | 330 (26%) | 0 (0%) | 330 (52%) |
| Atezolizumab + Carboplatin + nab-Paclitaxel | 431 (34%) | 431 (67%) | 0 (0%) |
| Bevacizumab + Carboplatin + Paclitaxel | 304 (24%) | 0 (0%) | 304 (48%) |
| Carboplatin + nab-Paclitaxel | 217 (17%) | 217 (33%) | 0 (0%) |
| BMI (kg/m^2^) | 25 (23 - 29) | 25 (23 - 29) | 25 (22 - 29) |
| WHO-defined BMI category | | | |
| normal | 590 (46%) | 291 (45%) | 299 (47%) |
| overweight | 455 (35%) | 234 (36%) | 221 (35%) |
| obesity I | 177 (14%) | 88 (14%) | 89 (14%) |
| obesity II | 45 (4%) | 27 (4%) | 18 (3%) |
| obesity III | 15 (1%) | 8 (1%) | 7 (1%) |
| Age | 64 (58 - 70) | 64 (58 - 71) | 63 (58 - 70) |
| Sex | | | |
| Female | 509 (40%) | 266 (41%) | 243 (38%) |
| Male | 773 (60%) | 382 (59%) | 391 (62%) |
| Race | | | |
| American Indian or Alaska Native | 2 (<1%) | 0 (0%) | 2 (<1%) |
| Asian | 73 (6%) | 15 (2%) | 58 (9%) |
| Black or African American | 38 (3%) | 24 (4%) | 14 (2%) |
| Multiple | 3 (<1%) | 1 (<1%) | 2 (<1%) |
| White | 1,127 (88%) | 583 (90%) | 544 (86%) |
| Missing | 39 (3%) | 25 (4%) | 14 (2%) |
| ECOG PS | | | |
| 0 | 531 (41%) | 266 (41%) | 265 (42%) |
| 1 | 745 (58%) | 381 (59%) | 364 (57%) |
| Missing | 6 (<1%) | 1 (<1%) | 5 (1%) |
| Tobacco history | | | |
| Current | 273 (21%) | 132 (20%) | 141 (22%) |
| Former | 846 (66%) | 455 (70%) | 391 (62%) |
| Never | 163 (13%) | 61 (9%) | 102 (16%) |
| Baseline tumour site count | | | |
| <3 | 803 (63%) | 286 (44%) | 517 (82%) |
| ≥3 | 479 (37%) | 362 (56%) | 117 (18%) |
| Baseline liver metastasis | 176 (14%) | 92 (14%) | 84 (13%) |
| Baseline bone metastasis | 202 (16%) | 182 (28%) | 20 (3%) |
| PD-L1 expression | | | |
| High | 246 (19%) | 126 (19%) | 120 (19%) |
| Low | 481 (38%) | 256 (40%) | 225 (35%) |
| Negative | 555 (43%) | 266 (41%) | 289 (46%) |
| Baseline lactate dehydrogenase (U/L) | | | |
| Median (IQR) | 236 (182 - 361) | 230 (179 - 373) | 241 (186 - 346) |
| Missing | 12 (1%) | 5 (1%) | 7 (1%) |
| Baseline neutrophil to lymphocyte ratio | | | |
| Median (IQR) | 4.1 (2.8 - 6.3) | 4.2 (2.9 - 6.2) | 4.1 (2.6 - 6.4) |
| Missing | 1 (0.1%) | 1 (0.2%) | 0 (0%) |
| Baseline albumin level (g/L) | | | |
| Median (IQR) | 39 (35 - 42) | 39 (36 - 42) | 39 (35 - 42) |
| Missing | 5 (<1%) | 1 (<1%) | 4 (1%) |
| Data are median (IQR) or number of patients (%). | | | |

Supplementary Table 2: Patient characteristic data by intervention type (chemoimmunotherapy and chemotherapy).

|  | **Total No. 1,282** | **Chemotherapy  No. 521** | **Chemoimmunotherapy No. 761** |
| --- | --- | --- | --- |
| Study | | | |
| IMPOWER130 | 648 (51%) | 217 (42%) | 431 (57%) |
| IMPOWER150 | 634 (49%) | 304 (58%) | 330 (43%) |
| Randomised Arm | | | |
| Atezolizumab + Bevacizumab + Carboplatin + Paclitaxel | 330 (26%) | 0 (0%) | 330 (43%) |
| Atezolizumab + Carboplatin + nab-Paclitaxel | 431 (34%) | 0 (0%) | 431 (57%) |
| Bevacizumab + Carboplatin + Paclitaxel | 304 (24%) | 304 (58%) | 0 (0%) |
| Carboplatin + nab-Paclitaxel | 217 (17%) | 217 (42%) | 0 (0%) |
| BMI | 25 (23 - 29) | 26 (23 - 29) | 25 (23 - 29) |
| WHO-defined BMI category | | | |
| normal | 590 (46%) | 232 (45%) | 358 (47%) |
| overweight | 455 (35%) | 189 (36%) | 266 (35%) |
| obesity I | 177 (14%) | 77 (15%) | 100 (13%) |
| obesity II | 45 (4%) | 17 (3%) | 28 (4%) |
| obesity III | 15 (1%) | 6 (1%) | 9 (1%) |
| Age | 64 (58 - 70) | 63 (58 - 70) | 64 (58 - 71) |
| Sex | | | |
| Female | 509 (40%) | 209 (40%) | 300 (39%) |
| Male | 773 (60%) | 312 (60%) | 461 (61%) |
| Race | | | |
| American Indian or Alaska Native | 2 (<1%) | 0 (0%) | 2 (<1%) |
| Asian | 73 (6%) | 23 (4%) | 50 (7%) |
| Black or African American | 38 (3%) | 18 (3%) | 20 (3%) |
| Multiple | 3 (<1%) | 0 (0%) | 3 (<1%) |
| White | 1,127 (88%) | 469 (90%) | 658 (86%) |
| Missing | 39 (3%) | 11 (2%) | 28 (4%) |
| ECOG PS | | | |
| 0 | 531 (41%) | 220 (42%) | 311 (41%) |
| 1 | 745 (58%) | 299 (57%) | 446 (59%) |
| Missing | 6 (<1%) | 2 (<1%) | 4 (1%) |
| Tobacco history | | | |
| Current | 273 (21%) | 118 (23%) | 155 (20%) |
| Former | 846 (66%) | 340 (65%) | 506 (66%) |
| Never | 163 (13%) | 63 (12%) | 100 (13%) |
| Baseline tumour site count | | | |
| <3 | 803 (63%) | 347 (67%) | 456 (60%) |
| ≥3 | 479 (37%) | 174 (33%) | 305 (40%) |
| Baseline liver metastasis | 176 (14%) | 71 (14%) | 105 (14%) |
| Baseline bone metastasis | 202 (16%) | 69 (13%) | 133 (17%) |
| PD-L1 expression | | | |
| High | 246 (19%) | 97 (19%) | 149 (20%) |
| Low | 481 (38%) | 188 (36%) | 293 (39%) |
| Negative | 555 (43%) | 236 (45%) | 319 (42%) |
| Baseline lactate dehydrogenase (U/L) | | | |
| Median (IQR) | 236 (182 - 361) | 238 (184 - 353) | 236 (181 - 362) |
| Missing | 12 (1%) | 6 (1%) | 6 (1%) |
| Baseline neutrophil to lymphocyte ratio | | | |
| Median (IQR) | 4.1 (2.8 - 6.3) | 4.1 (2.7 - 6.0) | 4.1 (2.9 - 6.5) |
| Missing | 1 (0.1%) | 1 (0.2%) | 0 (0%) |
| Baseline albumin level (g/L) | | | |
| Median (IQR) | 39 (35 - 42) | 39 (36 - 42) | 39 (35 - 42) |
| Missing | 5 (<1%) | 4 (1%) | 1 (<1%) |
| Data are median (IQR) or number of patients (%). | | | |

Supplementary Table 3: Patient characteristic data by BMI category among chemoimmunotherapy-treated cohort.

|  | **Total No. 761** | **Normal No. 358** | | **Overweight No. 266** | **Obese No. 137** |
| --- | --- | --- | --- | --- | --- |
| Study |  |  | |  |  |
| IMPOWER130 | 431 (57%) | 193 (54%) | | 163 (61%) | 75 (55%) |
| IMPOWER150 | 330 (43%) | 165 (46%) | | 103 (39%) | 62 (45%) |
| Randomised Arm |  |  | |  |  |
| Atezolizumab + Bevacizumab + Carboplatin + Paclitaxel | 330 (43%) | 165 (46%) | | 103 (39%) | 62 (45%) |
| Atezolizumab + Carboplatin + nab-Paclitaxel | 431 (57%) | 193 (54%) | | 163 (61%) | 75 (55%) |
| Age | 64 (58 - 71) | 64 (58 - 70) | | 64 (58 - 71) | 65 (58 - 71) |
| Sex |  |  | |  |  |
| Female | 300 (39%) | 141 (39%) | | 103 (39%) | 56 (41%) |
| Male | 461 (61%) | 217 (61%) | | 163 (61%) | 81 (59%) |
| Race |  |  | |  |  |
| American Indian or Alaska Native | 2 (<1%) | 1 (<1%) | | 1 (<1%) | 0 (0%) |
| Asian | 50 (7%) | 36 (10%) | | 14 (5%) | 0 (0%) |
| Black or African American | 20 (3%) | 8 (2%) | | 3 (1%) | 9 (7%) |
| Multiple | 3 (<1%) | 1 (<1%) | | 1 (<1%) | 1 (1%) |
| White | 658 (86%) | 297 (83%) | | 237 (89%) | 124 (91%) |
| Missing | 28 (4%) | 15 (4%) | | 10 (4%) | 3 (2%) |
| ECOG PS |  |  | |  |  |
| 0 | 311 (41%) | 145 (41%) | | 107 (40%) | 59 (43%) |
| 1 | 446 (59%) | 210 (59%) | | 158 (59%) | 78 (57%) |
| Missing | 4 (1%) | 3 (1%) | | 1 (<1%) | 0 (0%) |
| Tobacco history |  |  | |  |  |
| Current | 155 (20%) | 91 (25%) | | 41 (15%) | 23 (17%) |
| Former | 506 (66%) | 224 (63%) | | 189 (71%) | 93 (68%) |
| Never | 100 (13%) | 43 (12%) | | 36 (14%) | 21 (15%) |
| Baseline tumour site count |  |  | |  |  |
| <3 | 456 (60%) | 200 (56%) | | 154 (58%) | 102 (74%) |
| ≥3 | 305 (40%) | 158 (44%) | | 112 (42%) | 35 (26%) |
| Baseline liver metastasis | 105 (14%) | 51 (14%) | | 41 (15%) | 13 (9%) |
| Baseline bone metastasis | 133 (17%) | 71 (20%) | | 42 (16%) | 20 (15%) |
| PD-L1 expression |  |  | |  |  |
| High | 149 (20%) | 67 (19%) | | 55 (21%) | 27 (20%) |
| Low | 293 (39%) | 135 (38%) | | 101 (38%) | 57 (42%) |
| Negative | 319 (42%) | 156 (44%) | | 110 (41%) | 53 (39%) |
| Baseline lactate dehydrogenase (U/L) |  |  | |  |  |
| Median (IQR) | 236 (181 - 362) | 239 (183 - 361) | | 240 (180 - 387) | 223 (177 - 322) |
| Missing | 6 (1%) | 2 (1%) | | 3 (1%) | 1 (1%) |
| Baseline neutrophil to lymphocyte ratio | 4.1 (2.9 - 6.5) | 4.4 (2.9 - 7.0) | | 3.9 (2.9 - 6.4) | 3.8 (2.6 - 6.0) |
| Baseline albumin level (g/L) |  |  | |  |  |
| Median (IQR) | 39 (35 - 42) | 39 (35 - 42) | | 39 (35 - 42) | 40 (37 - 42) |
| Missing | 1 (<1%) | 1 (<1%) | | 0 (0%) | 0 (0%) |
| Data are median (IQR) or number of patients (%). |  | |  |  |  |

Supplementary Table 4: Patient characteristic data by BMI category among chemotherapy-treated cohort.

|  | **Total No. 521** | **Normal No. 232** | **Overweight No. 189** | **Obese No. 100** |
| --- | --- | --- | --- | --- |
| Study |  |  |  |  |
| IMPOWER130 | 217 (42%) | 98 (42%) | 71 (38%) | 48 (48%) |
| IMPOWER150 | 304 (58%) | 134 (58%) | 118 (62%) | 52 (52%) |
| Randomised Arm |  |  |  |  |
| Bevacizumab + Carboplatin + Paclitaxel | 304 (58%) | 134 (58%) | 118 (62%) | 52 (52%) |
| Carboplatin + nab-Paclitaxel | 217 (42%) | 98 (42%) | 71 (38%) | 48 (48%) |
| Age | 63 (58 - 70) | 63 (58 - 71) | 64 (58 - 71) | 63 (57 - 69) |
| Sex |  |  |  |  |
| Female | 209 (40%) | 105 (45%) | 66 (35%) | 38 (38%) |
| Male | 312 (60%) | 127 (55%) | 123 (65%) | 62 (62%) |
| Race |  |  |  |  |
| Asian | 23 (4%) | 15 (6%) | 5 (3%) | 3 (3%) |
| Black or African American | 18 (3%) | 7 (3%) | 7 (4%) | 4 (4%) |
| White | 469 (90%) | 203 (88%) | 174 (92%) | 92 (92%) |
| Missing | 11 (2%) | 7 (3%) | 3 (2%) | 1 (1%) |
| ECOG PS |  |  |  |  |
| 0 | 220 (42%) | 91 (39%) | 80 (42%) | 49 (49%) |
| 1 | 299 (57%) | 140 (60%) | 108 (57%) | 51 (51%) |
| Missing | 2 (<1%) | 1 (<1%) | 1 (1%) | 0 (0%) |
| Tobacco history |  |  |  |  |
| Current | 118 (23%) | 68 (29%) | 29 (15%) | 21 (21%) |
| Former | 340 (65%) | 138 (59%) | 136 (72%) | 66 (66%) |
| Never | 63 (12%) | 26 (11%) | 24 (13%) | 13 (13%) |
| Baseline tumour site count |  |  |  |  |
| <3 | 347 (67%) | 153 (66%) | 129 (68%) | 65 (65%) |
| ≥3 | 174 (33%) | 79 (34%) | 60 (32%) | 35 (35%) |
| Baseline liver metastasis | 71 (14%) | 25 (11%) | 30 (16%) | 16 (16%) |
| Baseline bone metastasis | 69 (13%) | 28 (12%) | 21 (11%) | 20 (20%) |
| PD-L1 expression |  |  |  |  |
| High | 97 (19%) | 42 (18%) | 34 (18%) | 21 (21%) |
| Low | 188 (36%) | 89 (38%) | 67 (35%) | 32 (32%) |
| Negative | 236 (45%) | 101 (44%) | 88 (47%) | 47 (47%) |
| Baseline lactate dehydrogenase (U/L) |  |  |  |  |
| Median (IQR) | 238 (184 - 353) | 235 (182 - 347) | 236 (182 - 374) | 246 (190 - 353) |
| Missing | 6 (1%) | 3 (1%) | 3 (2%) | 0 (0%) |
| Baseline neutrophil to lymphocyte ratio |  |  |  |  |
| Median (IQR) | 4.1 (2.7 - 6.0) | 4.3 (2.7 - 6.7) | 3.9 (2.6 - 5.4) | 4.1 (3.0 - 5.7) |
| Missing | 1 (0.2%) | 0 (0%) | 0 (0%) | 1 (1.0%) |
| Baseline albumin level (g/L) |  |  |  |  |
| Median (IQR) | 39 (36 - 42) | 38 (35 - 41) | 39 (36 - 43) | 39 (36 - 42) |
| Missing | 4 (1%) | 3 (1%) | 0 (0%) | 1 (1%) |
| Data are median (IQR) or number of patients (%). |  |  |  |  |
